# Prognosis and Characterization of Immune Microenvironment in Acute Myeloid Leukemia Through Identification of an Autophagy-Related Signature

**DOI:** 10.3389/fimmu.2021.695865

**Published:** 2021-05-31

**Authors:** Denggang Fu, Biyu Zhang, Shiyong Wu, Yinghua Zhang, Jingwu Xie, Wangbin Ning, Hua Jiang

**Affiliations:** ^1^ Department of Pediatrics, The Wells Center for Pediatric Research, Indiana University School of Medicine, Indianapolis, IN, United States; ^2^ School of Pharmacy and Life Science, Jiujiang University, Jiujiang, China; ^3^ The IU Simon Comprehensive Cancer Center, Indiana University, Indianapolis, IN, United States; ^4^ Department of Rheumatology and Immunology, Xiangya Hospital, Central South University, Changsha, China

**Keywords:** acute myeloid leukemia, autophagy, signature, tumor immune microenvironment, prognosis

## Abstract

Acute myeloid leukemia (AML) is one of the most common hematopoietic malignancies that has an unfavorable outcome and a high rate of relapse. Autophagy plays a vital role in the development of and therapeutic responses to leukemia. This study identifies a potential autophagy-related signature to monitor the prognoses of patients of AML. Transcriptomic profiles of AML patients (GSE37642) with the relevant clinical information were downloaded from Gene Expression Omnibus (GEO) as the training set while TCGA-AML and GSE12417 were used as validation cohorts. Univariate regression analyses and multivariate stepwise Cox regression analysis were respectively applied to identify the autophagy-related signature. The univariate Cox regression analysis identified 32 autophagy-related genes (ARGs) that were significantly associated with the overall survival (OS) of the patients, and were mainly rich in signaling pathways for autophagy, p53, AMPK, and TNF. A prognostic signature that comprised eight ARGs (BAG3, CALCOCO2, CAMKK2, CANX, DAPK1, P4HB, TSC2, and ULK1) and had good predictive capacity was established by LASSO–Cox stepwise regression analysis. High-risk patients were found to have significantly shorter OS than patients in low-risk group. The signature can be used as an independent prognostic predictor after adjusting for clinicopathological parameters, and was validated on two external AML sets. Differentially expressed genes analyzed in two groups were involved in inflammatory and immune signaling pathways. An analysis of tumor-infiltrating immune cells confirmed that high-risk patients had a strong immunosuppressive microenvironment. Potential druggable OS-related ARGs were then investigated through protein–drug interactions. This study provides a systematic analysis of ARGs and develops an OS-related prognostic predictor for AML patients. Further work is needed to verify its clinical utility and identify the underlying molecular mechanisms in AML.

## Introduction

AML is one of the most aggressive blood malignancies that is characterized by a heterogeneity of molecular abnormalities and the accumulation of immature myeloid progenitors in the bone marrow and peripheral blood (1, 2). An estimated 19,940 new cases of AML were diagnosed in the US in 2020, with 11,180 deaths (3). The mainstream treatment for AML patients is chemotherapy, but most patients relapse or succumb to the disease after initial remission. Although extensive efforts have been made to develop targeted therapy and/or combined therapy for it (4), the 5-year survival rate of patients of AML is still less than 30%. Thus, it is critical to identify novel prognostic biomarkers to monitor patients’ prognoses and better understand the pathogenesis of AML.

Autophagy is a complex multistep self-digestive cellular process that is essential for the survival, differentiation, and homeostasis of cells (5). It sequesters damaged organelles/proteins, invading pathogens, and macromolecules in an autophagosome coated with a double membrane. Following the fusion of the autophagosome with lysosome, these materials are degraded to maintain the recycling balance between the synthesis and the consumption of the cellular components (6). In normal conditions, autophagous activity is too low to require essential nutrients of the cell by removing unfolded and excessively aged proteins, while the dysregulation of autophagy is involved in a diversity of pathologies, including tumorigenesis, infections, aging, and heart disease (7). Autophagy can be a double-edged sword for organisms in that it can prevent the formation of tumors but can also promote the survival and proliferation of cancer cells by providing them with nutrients (8). A variety of roles of autophagy have been identified in hematopoietic disease. It is required for maintaining the functions of hematopoietic stem cells (9) and T-lymphoid lineages (10, 11), and for responses to extracellular cytokine stimuli (12). Increasing evidence has shown that autophagy is a key mechanism in leukemogenesis and chemoresistance, and this has made it an attractive therapeutic target in research in recent years (13, 14). A number of autophagy-inducing agents, such as arsenic trioxide, vitamin D3, eupalinin A, APO866, and platonin, have been developed to initiate the death of leukemic cells (15). A variety of essential genes are involved in the machinery of autophagy to control the balance of catabolic processes (16). Research on the role of autophagy in the progression of AML and responses to the treatment of patients has focused on one or more autophagy-related genes (ARGs) (15), and few studies have sought to systematically clarify the potential roles of expressions of these ARGs in predicting the prognoses of AML patients.

In this study, we identified survival-related ARGs in the context of AML and develop a prognostic signature for AML patients to profile their expressions. Transcriptomic datasets of AML were downloaded from publicly accessible databases, and were divided into training and validation sets. Univariate Cox regression analysis was used to assess the prognostic effects of these ARGs for AML. Least absolute shrinkage and selection operator (LASSO) Cox regression were performed to determine the key variables and construct an ARG-related risk signature for the AML patients. The predictive accuracy of the risk signature was analyzed on the validation set, and the results suggest that it is an effective predictor of patient outcomes that is independent of the clinical parameters used to monitor them. The abundance of tumor-infiltrating immune cells defined by the signature reflected the distinct microenvironmental landscape of the tumor, and potential druggable ARGs were identified. A general analysis workflow is diagrammed in **Figure S1**.

## Materials and Methods

### Data Collection and Processing

The transcriptomic profiles of three AML cohorts along with detailed clinicopathological information on them were downloaded from public databases. Raw microarray datasets of GSE37642 (17) and GSE12417 (18) were downloaded from the GEO (https://www.ncbi.nlm.nih.gov/geo/) and normalized by the robust multiarray average (RMA) algorithm using the affy package (19) between arrays. Batch effects were removed by the combat algorithm in the sva package (20). The AML RNA-seq dataset was downloaded from the UCSC Xena database (https://xenabrowser.net/datapages/). The available clinical information of samples used in this study was shown in **Table S1**.

### Acquisition of ARGs

A total of 232 autophagy-related genes (ARGs) were derived from the Human Autophagy Database (HADb, http://autophagy.lu/clustering/index.html). The HADb provides a complete and an up-to-date list of human genes and proteins involved in the biological processes of autophagy reported in the literature (21). A total of 187 ARGs were available in the expression profiles obtained from GSE37642 (**Table S2**).

### Identification of Overall Survival (OS)-Related ARGs

The GSE37642 (n=553) was used as the training set to clarify the potential prognostic significance of these ARGs in the AML patients. OS-related ARGs with *P* < 0.05 were identified using univariate Cox hazard regression analysis.

### Functional Enrichment Analysis of OS-Related ARGs

Functional enrichment analysis, including gene ontology (GO) and the Kyoto Encyclopedia of Genes and Genomes (KEGG), was performed to unravel the main functions of OS-related ARGs in AML by using the clusterProfiler package (22). The Benjamin–Hochberg adjusted *P* < 0.05 was regarded as statistically significant.

### Molecular Characteristics of OS-Related ARGs

To investigate potential regulatory interactions among these ARGs, a protein–protein interaction (PPI) network was formulated using the STRING database (23) and displayed in Cytoscape (version 3.8.0) (24). To identify the hub modules in the network, the Molecular Complex Detection (MCODE) plugin (25) in Cytoscape was used to extract densely connected modules with the default parameters “Degree Cutoff = 2,” “Node Score Cutoff = 0.2,” “K-Core = 2,” and “Max.Depth = 100.” The CytoNCA plugin (26) was used to calculate the nodes with the highest degree scores.

The key regulatory factors (TFs) of these OS-related ARGs were identified using the Transcriptional Regulatory Relationships Unraveled by Sentence-based Text mining (TRRUST) database, which is an online tool curated to explore transcriptional regulatory interactions in humans and mice (27).

### Construction and Validation of ARG-Related Prognostic Signature for AML Patients

To avoid overfitting the prognostic risk signature, we used the least absolute shrinkage and selection operator (LASSO)-based Cox regression (28) on the training dataset to identify the most significant features within the OS-related ARGs. These candidates were subjected to a multivariate Cox proportional hazards regression with the stepwise selection of variables based on the Akaike information criterion (29). The risk score of final optimized prognostic signature was calculated as follows:

$$\text{Risk score=}\sum_{i}^{n}Coefi\times {\text{A}}_{\text{i}}$$

where Coef is the regression coefficient, “i” represents the ARG that comprised of the signature, A represents the relative value of the expression of the individual ARG in the signature, and n represents the number of genes in the signature. The patients were divided into high- and low-risk groups based on median risk score as cutoff value. The differences in the OS of patients were assessed by Kaplan–Meier analysis and the log-rank test. The time-dependent receiver operating characteristic (ROC) curve (30) was employed to evaluate the predictive capacity of the ARG-based signature.

To test the predictive accuracy of the signature, two external AML cohorts—TCGA-LAML (n=149) and GSE12417 (n=242)—were downloaded and used as validation sets. The risk score for each patient was calculated by using the signature, and the Kaplan–Meier curve was used to reflect its discrimination-related performance.

### Identification and Enrichment Analysis of Differentially Expressed Genes (DEGs)

The differentially expressed genes (DEGs) between the high- and low-risk groups were identified using the limma package (31). To better understand the functions of the DEGs in AML, we used the clusterProfiler package (22) for enrichment analysis, including the GO terms, including biological process (BP), molecular function (MF) and cellular component (CC), and KEGG pathways. The DEGs were clustered and a heatmap for them was generated *via* ClustVis (32).

### Gene Set Enrichment Analysis (GSEA)

The patients were divided into high- and low-risk groups according to the median risk score, as mentioned above. GSEA was performed to identify the primarily enriched pathways using GSEA 4.02 (http://www.broad.mit.edu/gsea/) (33). Pathway with the nominal *P* < 0.05 and FDR < 0.25 were considered statistically significant.

### Development of Autophagy Clinicopathologic Nomogram

To predict the OS of each AML patient, an autophagy clinicopathologic nomogram that incorporated the prognostic signature into the clinicopathologic parameters available in the training set was conducted through the rms package (34). The final nomogram was extracted using the Akaike information criterion (AIC) for variable selection. The calibration curve was used to assess the predictive discrimination of the signature for AML patients (35).

### Tumor-Immune Microenvironment Landscape and Potential Implications for Immunotherapy Defined by the Signature

CIBERSORT was used to calculate the abundance of infiltration of 22 immune cell types within a complex mixture of the gene expression data of the AML patients (36), including seven types of T cells, naïve and memory B cells, plasma cells, and NK cells, in the high- and low-risk groups. Samples with *P* < 0.05 were chosen for further analysis.

Recent years have witnessed a rise in immunotherapy and targeted therapy for AML patients. We predict the potential effect of treatment according to risk score here by analyzing the correlation between risk score and therapeutic targets in clinical trials or clinical practice using Pearson’s correlation analysis (37, 38). The targets of therapy were as follows: programmed cell death ligand (PD-1), ASXL1, BCL2, CD33, CD47, CHEK1, PLK1, DOT1L, FMS-like tyrosine kinase 3 (FLT3), Cytotoxic T-Lymphocyte-associated Protein 4 (CTLA4), IDH1, IDH2, MCL1, and MDM2.

To find potential drug targets, protein–drug interactions were analyzed in the survival-related ARGs using NetworkAnalyst 3.0 (https://www.networkanalyst.ca/). Information on the protein and drug contents of the targets were retrieved from DrugBank (version 5.0, https://go.drugbank.com/) (39).

## Results

### Identification and Functional Enrichment Analysis of OS-Related ARGs in AML

**Figure 1:**
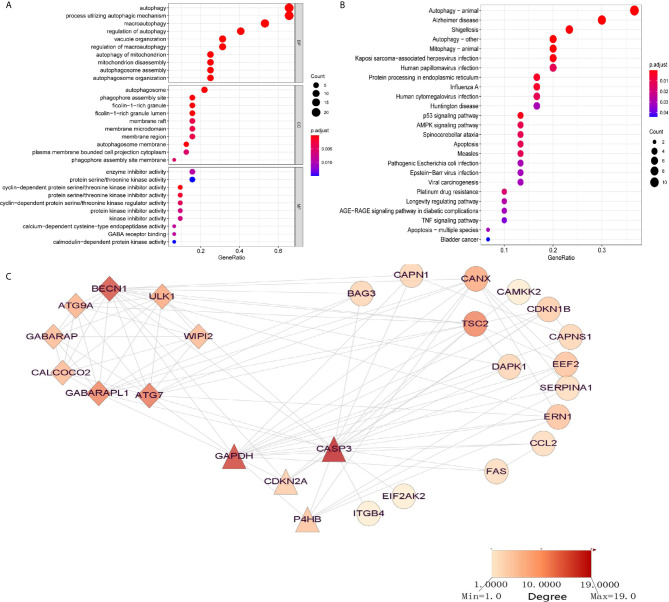
Significantly enriched GO terms and KEGG pathways of OS-related autophagy-related genes (ARGs) (adjusted *P* < 0.05). **(A)** Significantly enriched GO terms of OS-related ARGs. **(B)** Significantly enriched pathways of OS-related ARGs. **(C)** Two modules (CASP3 and BECN1 modules) identified through protein–protein interaction network analysis of OS-related ARGs. The color of the node in each module reflects its degree score.

To discover the potential prognostic significance of each available ARG in the AML training set, a univariate Cox proportional hazard regression analysis was used to screen out ARGs with a *P*-value less than 0.05. The expressions of 32 ARGs were thus found to be significantly associated with the OS of the AML patients (**Table 1**). GO functional analysis of the OS-related ARGs showed that they were primarily active in processes that utilized autophagy-related mechanisms (**Figure 1A**). These ARGs were involved in autophagy, human cytomegalovirus infection, the p53 signaling pathway, AMPK signaling pathway, and apoptosis (**Figure 1B**).

**Table 1:** Overall survival-related ARGs in the AML patients (*P* < 0.05).

Gene symbol	HR (95% CI)	*P* value
TSC2	0.4072(0.2832 - 0.5856)	1.25E-06
CALCOCO2	0.6758(0.5680 - 0.8039)	9.69E-06
DAPK1	1.3835(1.1847 - 1.6157)	4.10E-05
BAG3	1.1503(1.0753 - 1.2305)	4.72E-05
CAMKK2	0.5397(0.3882 - 0.7504)	0.000244719
CANX	0.7301(0.6099 - 0.8739)	0.000606334
ULK1	0.7257(0.6025 - 0.8742)	0.000735293
P4HB	0.8215(0.7324 - 0.9215)	0.000790605
CCL2	1.1482(1.0510 - 1.2544)	0.002205099
GABARAP	0.7085(0.5667 - 0.8859)	0.002507313
GABARAPL1	0.8216(0.7213 - 0.9360)	0.003127288
EEF2	0.6578(0.4920 - 0.8793)	0.004684677
GAPDH	0.7715(0.6369 - 0.9345)	0.007994922
NCKAP1	1.6932(1.1345 - 2.5271)	0.009954145
CDKN1B	1.1991(1.0424 - 1.3793)	0.011027097
CAPNS1	0.8504(0.7469 - 0.9684)	0.014474617
SERPINA1	0.9062(0.8370 - 0.9811)	0.015041995
BECN1	0.7031(0.5283 - 0.9358)	0.015733367
ARSB	0.7888(0.6501 - 0.9571)	0.016217681
CDKN2A	0.7312(0.5641 - 0.9477)	0.017994775
DLC1	0.7460(0.5785 - 0.9619)	0.023876677
ERN1	0.6722(0.4728 - 0.9558)	0.026995358
CAPN1	0.8331(0.7073 - 0.9813)	0.028824089
ATG9A	0.8343(0.7059 - 0.9861)	0.03365361
EIF2AK2	1.2624(1.0181 - 1.5653)	0.03374212
WIPI2	0.7297(0.5453 - 0.9765)	0.034038805
ITGB4	0.5864(0.3552 - 0.9682)	0.036949174
CLN3	0.7788(0.6128 - 0.9897)	0.04089584
ATG7	0.8389(0.7072 - 0.9952)	0.043928123
HGS	0.8035(0.6488 - 0.9951)	0.044918193
FAS	0.8763 (0.7692 - 0.9982)	0.046832376
CASP3	0.8846(0.7838 - 0.9983)	0.046888189

To discover interactions among these OS-related ARGs, two significant modules were identified, using PPI network analysis, with more than four nodes: CASP3 and BECN1 (**Figure 1C**). The BECN1 module contained eight nodes with 28 edges, whereas GAPDH, CDKN2A, and P4HB were the three nodes of the CASP3 module. These ARGs might have important implications for the pathogenesis of AML.

To identify the transcriptional regulators of the OS-related ARGs, 16 TFs were identified in the TRRUST database (**Table S3**), including nuclear transcription factors (NFYC, NFYB, NFYA, SP1, HSF1, E2F1), a signal transducer and an activator of transcription (STAT1, STAT3), TP53, and key members of NF-κB signaling (NFKB1, RELA). The gene expressions of several ARGs were significantly regulated, such as those of the important nodes CASP3, BECN1, ATG7, BAG3, and UKL1.

### Development and Validation of ARG-Related Prognostic Signature

**Figure 2:**
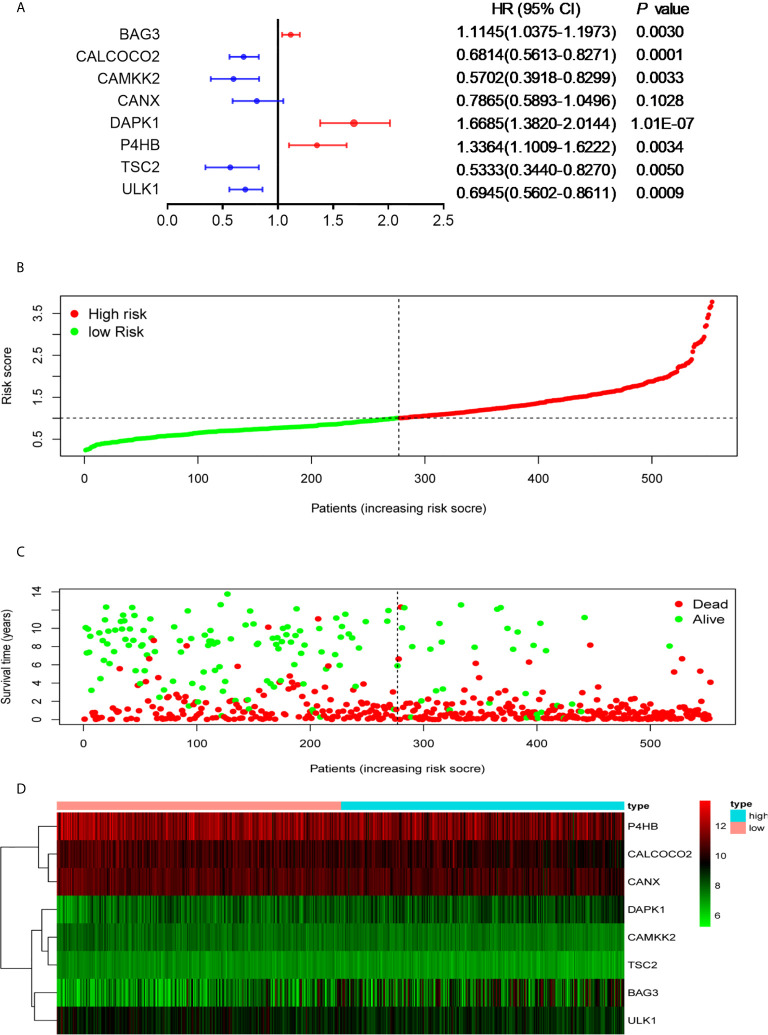
Development of the prognostic signature based on OS-relevant ARGs. **(A)**. The hazard ratio of model genes. **(B)** Distribution of the patients’ risk scores. **(C)** Patients’ survival times along with their risk scores. **(D)** The expressions of the eight model genes in the high- and low-risk groups.

To avoid potential overfitting, LASSO Cox regression analysis was used to select the key OS-related ARGs for modeling (**Figures S2A, B**). Eight ARGS were identified and used to develop an optimal prognostic signature for the OS of patients by multivariate Cox proportional hazards regression analysis by using forward and backward algorithms (**Figure 2A**). The patients’ risk scores were defined as follows:

$$\begin{array}{l} \text{Risk score=}\left[\text{Expression level of BAG}3*(0.1084)\right]+\left[\text{Expression level of CALCOCO}2\;*\;(-0.3836)\right]\\ +\left[\text{Expression level of CAMKK2 }*\;(-0.5617)\right]+\left[\text{Expression level of CANX }*\;(-0.2402)\right]\\ +\left[\text{Expression level of DAPK}1*(0.5119)\right]+\left[\text{Expression level of P}4\text{HB}*(0.2899)\right]\\ +\left[\text{Expression level of TSC}2*(-0.6286)\right]+\left[\text{Expression level of ULK}1*(-0.3645)\right] \end{array}$$

**Figure 3:**
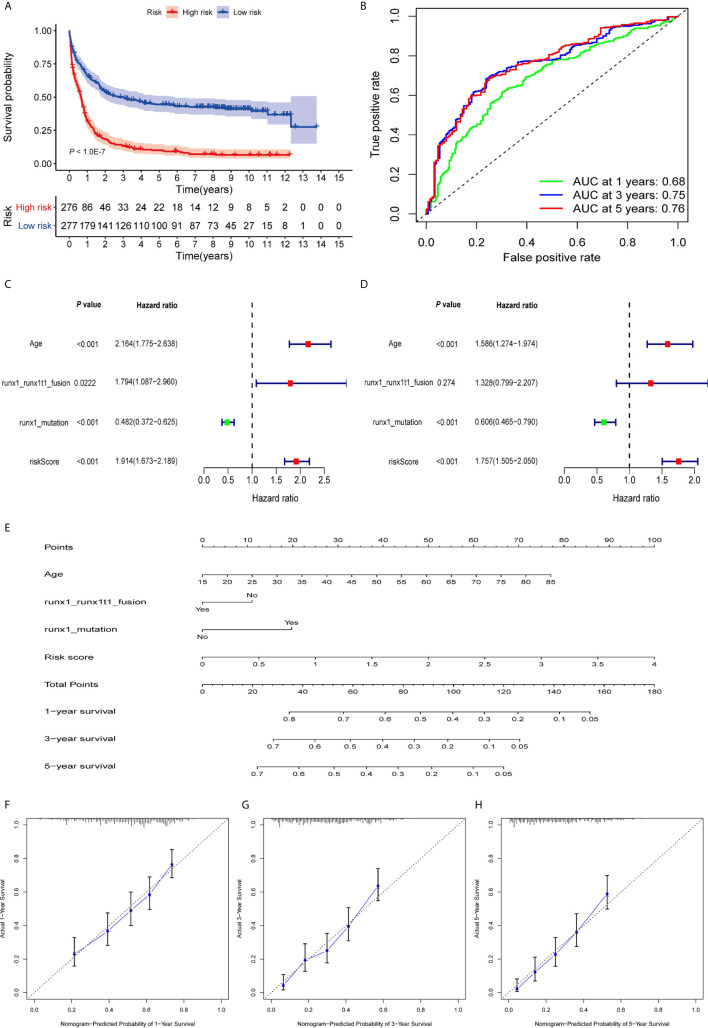
Evaluation of prognostic signature to predict the OS of AML patients. **(A)**. Patients in the high-risk group had significantly shorter OS than those in the low-risk group. **(B)** The AUC curves of the signature for 1, 3, and 5 years. **(C)** Univariate Cox regression analysis of the risk scores and clinical parameters. **(D)** Multivariate Cox regression analysis of the risk scores and clinical parameters. **(E)** Development of autophagy clinicopathologic nomogram for predicting 1-, 3-, and 5-year OS for AML patients by incorporating risk score, age, runx1 and runx1t1 fusion, and mutations in runx1. **(F–H)** Calibration curves of the autophagy clinicopathologic nomogram-predicted and observed 1-, 3-, and 5-year survival of AML patients. The dashed line represents the ideal performance, and the actual performance of the signature is represented by the blue lines.

The patients were divided into high- and low-risk groups according to the median value of risk score. As the risk scores of patients increased in both groups, the number of deaths increased (**Figures 2B, C**). With regard to expressions of the eight ARGs, BAG3 and DAPK1 were highly expressed in the high-risk group (**Figures 2D**, **S3A, B**), and CALCOCO2, CAMKK2, CANX, P4HB, TSC2, and ULK1 were expressed high in the low-risk group (**Figures 2D**, **S3C–H**). This is consistent with evidence that blasts in AML show reduced expressions for most ARGs, indicating that low autophagy-related activity promotes leukemic development (40). To determine the predictive performance of the signature, the Kaplan–Meier analysis showed that patients in the high-risk group had significantly shorter OS than patients in the low-risk group (*P* < 1.0E-07, **Figure 3A**). To assess the predictive accuracy of the signature, the AUC of our signature for a 5-year OS was 0.76. In addition, the AUCs for 1-year and 3-year OS were 0.68 and 0.75, respectively, and indicated high predictive capacity of the signature (**Figure 3B**).

To calculate the predictive independence of the signature for AML patients, univariate Cox regression analysis showed that age, runx1 and runx1t1 fusion, mutations in runx1, and risk score were significantly correlated with the OS of the patients (**Figure 3C**). The multivariate Cox regression analysis showed that the risk score was an independent predictor for AML patients after adjusting for these clinical parameters (**Figure 3D**), although age and mutations in runx1 were also independent. A comparison of the capability of OS predictions for AML patients based on the risk score and clinical factors showed that the AUCs of 1-year, 3-year, and 5-year OS of the clinical variables were inferior to those patients of the risk scores (**Figures S4A–C**).

For a more accurate evaluation of the signature, a nomogram that integrated the risk score, age, runx1_runx1t1 fusion, and runx1 mutations, was constructed (**Figure 3E**). The calibration curves showed that it could accurately predict the utility of 1-year, 3-year, and 5-year OS for AML patients (**Figures 3F–H**). This indicates that combining our risk scores and the clinical variables can improve OS prediction.

### Gene Set Enrichment Analysis

The distinct OS rates of patients in the high- and low-risk group were observed, and GSEA was used to investigate the potential molecular functional difference between them. mTOR-related signaling, AKT1 signaling, and relapse prognosis for AML relapse were significantly abundant in the low-risk group (**Figure S5**). Previous studies have shown that mTOR regulates cell growth and proliferation by controlling the biological processes of mRNA translation, autophagy, and metabolism, or dual interactions with AKT family signaling to activate or deactivate mTOR-dependent processes (41). These data highlight that autophagy-related events were mainly implicated in low-risk AML patients.

### Validation of Prognostic Signature in External AML Cohorts

**Figure 4:**
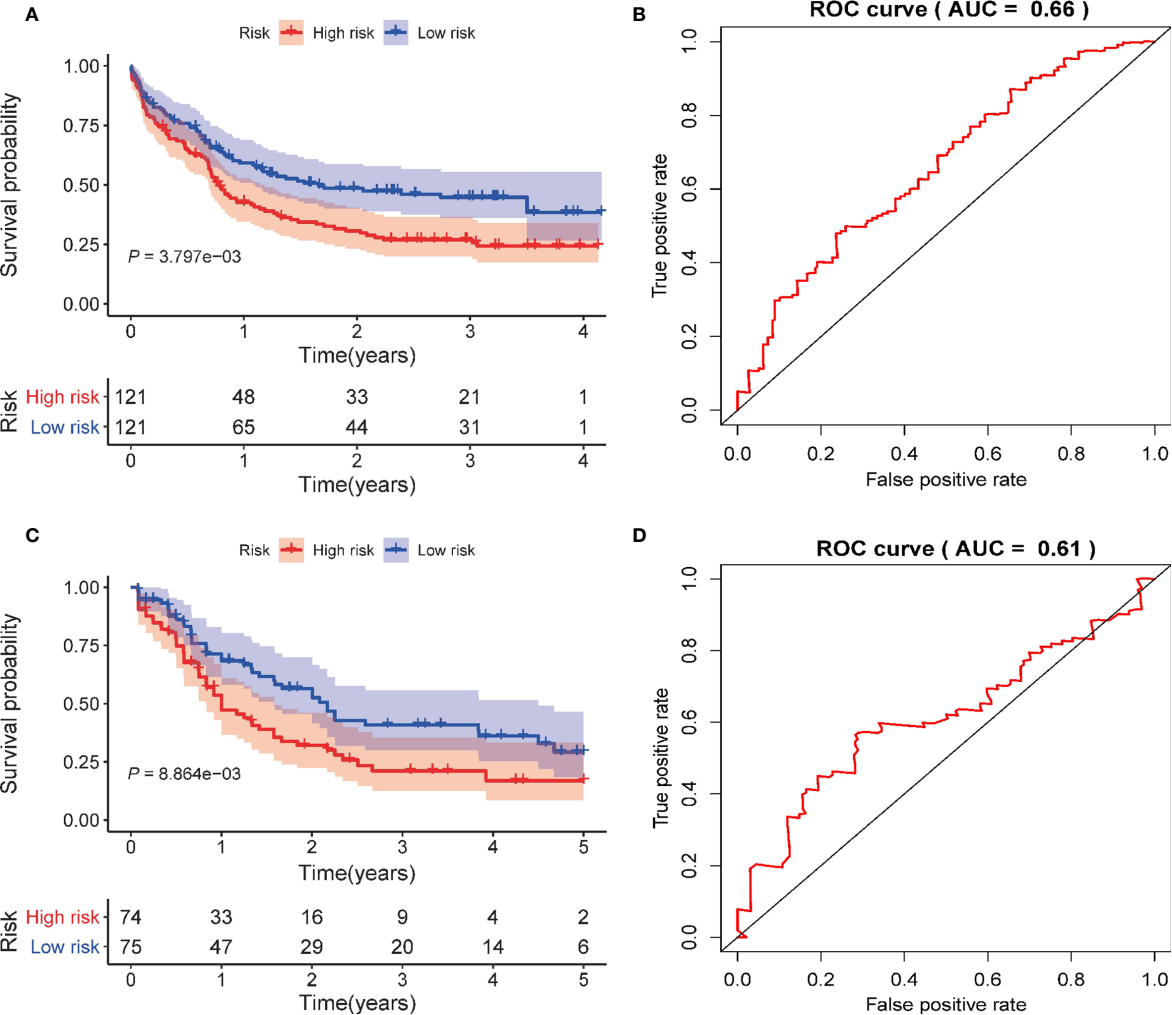
Validation of the autophagy-related prognostic signature on external AML cohorts. **(A)** Kaplan–Meier curve of the prognostic signature in the GSE12417 cohort. **(B)** The AUC curve of the signature for 3 years in the GSE12417 cohort. **(C)** Kaplan–Meier curve of the prognostic model in the TCGA cohort. **(D)** The AUC curve of the signature for 3 years in the TCGA cohort.

To test the predictive utility of the prognostic signature of the patients’ OS in the external AML cohorts (GSE12417 and TCGA-LAML), the risk score for each patient was calculated based on the formula for the signature. The patients were divided into high- and low-risk groups according to median risk score. The OS times of patients in the high-risk group were significantly shorter than patients in the low-risk group (*P* = 3.797E-03, **Figure 4A**) in the GSE12417 cohort. The AUC of the 3-year OS for this cohort was 0.66 (**Figure 4B**). In addition, the prognosis of patients in the high-risk group was worse than that of patients in the low-risk group in the TCGA-LAML set (*P* = 8.864E-03, **Figure 4C**). Similarly, the AUC of the 3-year OS was 0.612 (**Figure 4D**). Overall, these data show that the signature could be used to independently predict the OS for AML patients.

### Identification and Enrichment of Differentially Expressed Genes (DEGs)

**Figure 5:**
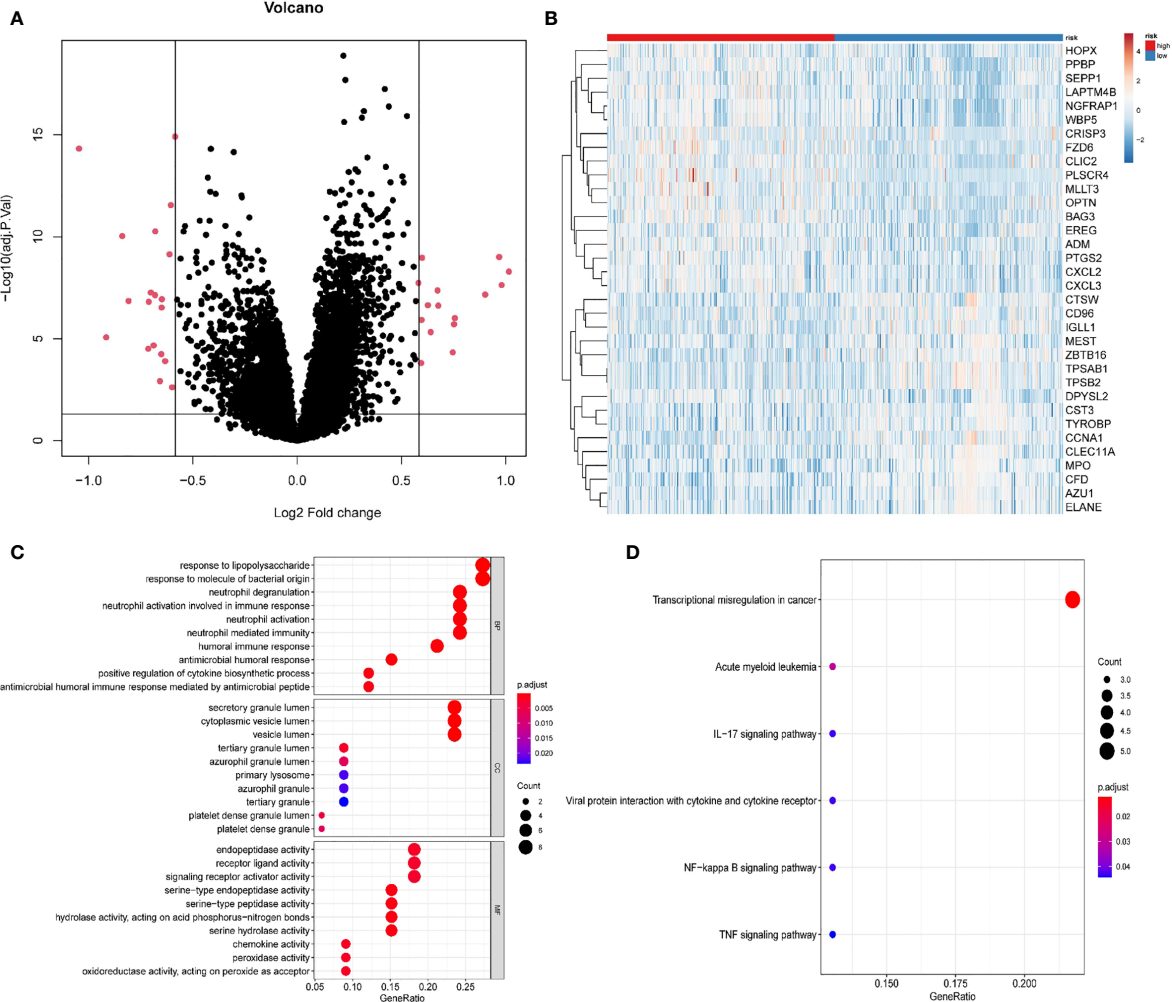
Differentially expressed genes (DEGs) between the high-risk and the low-risk groups. **(A)** Volcano plot of the DEGs. **(B)** Heatmap of the DEGs. **(C)** Significantly enriched GO terms of the DEGs. **(D)** Significantly enriched pathways of the DEGs.

We noted the differences in OS between patients in the high- and low-risk groups. To delineate the DEGs of the two groups, 34 DEGs were identified using the limma package, with 15 up-regulated genes and 19 down-regulated genes (**Figure 5A**). A distinct pattern of gene expression was observed in patients in the high- and low-risk groups (**Figure 5B**). The GO term analysis showed that these DEGs were significantly involved in the biological processes of neutrophil-related activities (activation, degranulation, and response to immunity), the cellular components that occur in secretory and cytoplasmic lumen and lysosome, and various peptidase activities (**Figure 5C**). The pathways referenced from the KEGG database showed that the DEGs highly expressed in the high-risk group were mainly involved in acute myeloid leukemia while the DEGs down-regulated in the low-risk group were markedly involved in signaling pathways for IL-17, viral protein interactions with cytokine and cytokine receptor, NF-kappa B signaling, and the TNF signaling pathway (**Figure 5D**). The data indicate that these DEGs might play important roles in AML progression and immune response.

### Potential Relevance of Signature in Tumor-Immune Microenvironment

**Figure 6:**
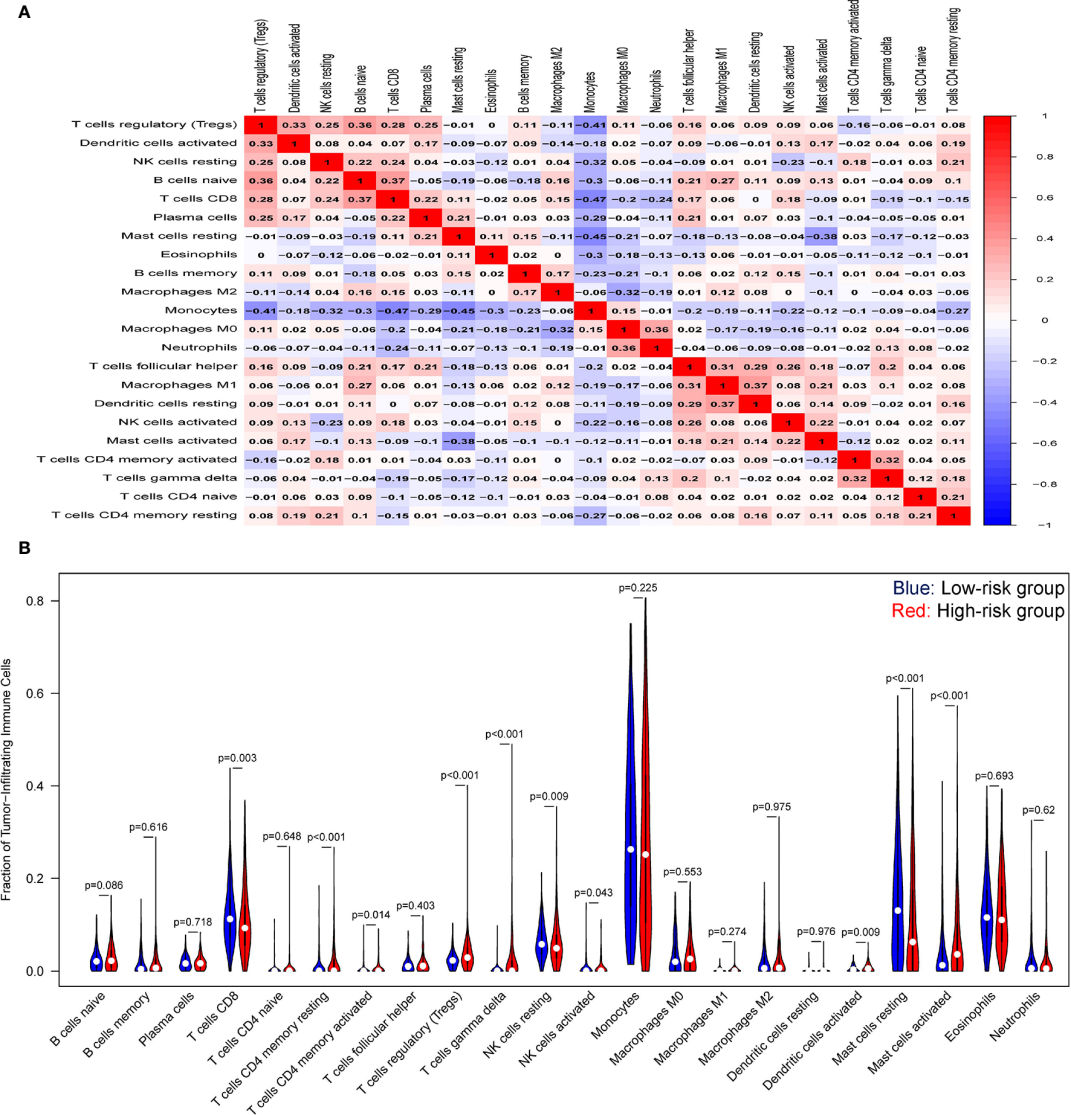
Tumor-immune microenvironment analysis of the high- and low-risk groups. **(A)** Correlation heatmap of the ratio of tumor-infiltrating immune cells. **(B)** Difference between tumor-infiltrating immune cells. The blue violin reflects the low-risk group and the red violin represents the high-risk group.

Tumor-infiltrating lymphocytes (TILs) in the tumor microenvironment (TME) are involved in cancer progression, drug resistance, and clinical outcomes. As displayed in **Figure 6**, an analysis of immune cell-infiltration in the TME as defined by our signature in training set showed that CD8 T cells, resting and activated NK cells, monocytes, and mast resting cells had significantly increased in the low-risk group, while CD4 T memory resting and activated cells, T cells gamma delta, regulatory T cells, and dendritic cells had been activated at high levels in the high-risk patients. Similar trends of tumor immune infiltration have been found in the two external validation sets (**Figure S6**). In addition, the expression analysis of exhausted cytotoxicity T cells markers indicated that GZMB and Interferon gamma are significantly increased in patients in high-risk group than those patients in low-risk group (**Figure S7**). This suggests a strong immunosuppressive TME that might weaken the capacity to defend against cancer in the high-risk group.

**Figure 7:**
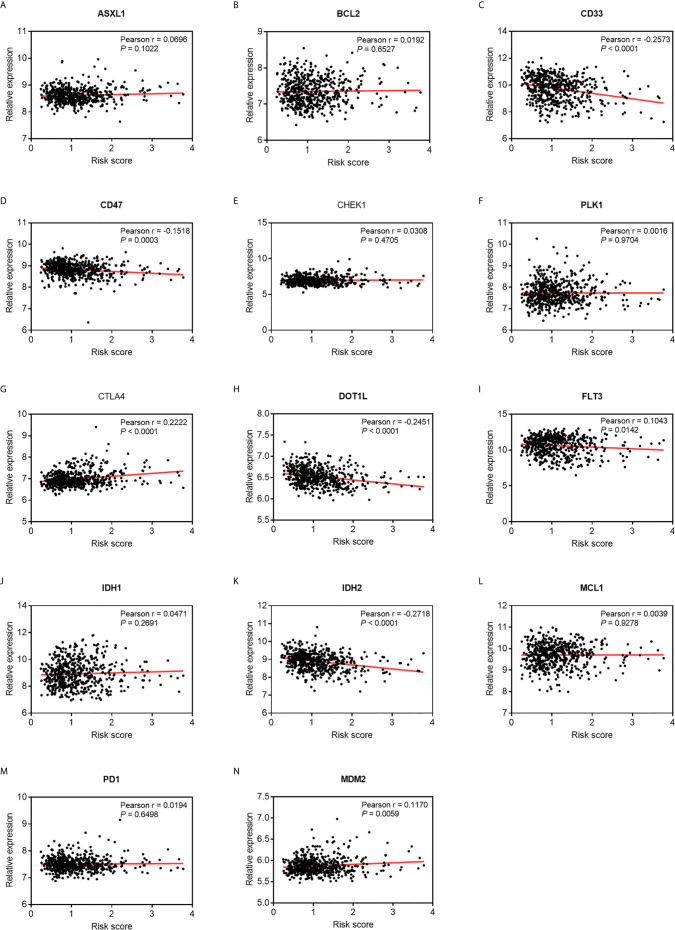
Pearson correlation of the risk scores of the targets of immunotherapy and targeted therapy. **(A)** ASXL1. **(B)** BCL2. **(C)** CD33. **(D)** CD47. **(E)** CHEK1. **(F)** PLK1. **(G)** CTLA4. **(H)** DOT1L. **(I)** FLT3. **(J)** IDH1. **(K)** IDH2. **(L)** MCL1. **(M)** PD-1. **(N)** MDM2.

Emerging molecules for immunotherapy and targeted therapy, such as immune checkpoint inhibitors, were recently identified and tested in pre- or clinical trials for the treatment of patients with AML. As shown in **Figure 7**, a Pearson correlation analysis showed that the risk score was significantly negatively correlated with the mRNA expressions of CD33 (cor = -0.2573, *P* < 0.0001), CD47 (cor = -0.1518, *P* = 0.0003), DOT1L (cor = -0.2451, *P* < 0.0001), and IDH2 (cor = -0.2718, *P* < 0.0001), and was positively related with those of CTLA4 (cor = 0.2222, *P* < 0.0001), FLT3 (cor = 0.1043, *P* = 0.0142), and MDM2 (cor = 0.1170, *P* = 0.0059). This suggests that patients with high risk scores might better respond to therapies targeting CTLA4, FLT3, and MDM2.

### Multiple Survival-Related ARGs Are Potential Druggable Targets

To determine whether any of the available survival-related ARGs were druggable targets, a protein–drug interaction analysis of these ARGs was conducted through NetworkAnalyst 3.0, using data from the DrugBank database. The protein products of eight ARGs were identified as drug targetable (**Table 2**). A majority of these ARGs have been demonstrated to be implicated in tumorigenesis, including CASP3 (Caspase 3) (42), EEF2 (eukaryotic translation elongation factor 2) (43), GAPDH (glyceraldehyde-3-phosphate dehydrogenase) (44), CAPN1 (Calpain 1) (45), DAPK1 (death-associated protein kinase 1) (46), SERPINA1 (serpin family A member 1) (47), and CCL2(C-C motif chemokine ligand 2) (48). Caspase-3 controlled AML1-ETO-induced leukemogenesis through autophagy modulation in a ULK1-dependent pattern, which indicates that the balance and selectivity among its substrates regulated disease progression (42). Eleven candidate drugs targeting Caspase-3 were identified. Caspase-3 inhibitors may be carboxylic acids and derivatives, such as 2-hydroxy-5-(2-mercapto-ethylsufamoyl)-benzoic acid and (1S)-2-oxo-1-phenyl-2-[(1,2,3,4-tetrahydroisoquinolin-5-yl)amino]ethyl acetate.

**Table 2:** Eight OS-related ARGs targeted by the drugs available from DrugBank.

Gene Symbol	HR (95%CI)	*P*-value	Number of drugs	DrugBank ID	Drug name examples
CASP3	0.8846(0.7838-0.9983)	0.04689	11	DB08497, DB08229, DB08213, DB07696, DB01017, DB06862, DB05408, DB03124, DB08498, DB08251, DB08499	EMRICASAN, MINOCYCLINE, METHYL (3S)-3-[(TERT-BUTOXYCARBONYL]-4-OXOPENTANOATE, 2-HYDROXY-5-(2-MERCAPTO-ETHYLSUFAMOYL)-BENZOIC ACID
EEF2	0.6578(0.4920-0.8793)	0.00468	4	DB04315,DB03223,DB08348,DB02059	Diphthamide, Glycinamide, Guanosine-5’-Diphosphate, Adenosine-5-Diphosphoribose
GAPDH	0.7715(0.6369-0.9345)	0.00799	4	DB03893,DB00157,DB07347	Thionicotinamide-Adenine-Dinucleotide,NADH,4-(2-Aminoethyl)Benzenesulfonyl Fluoride
CAPN1	0.8331(0.7073-0.9813)	0.02882	3	DB07627,DB04276,DB04653	CBZ-LEU-LEU-TYR-CH2F
DAPK1	1.3835(1.1847-1.6157)	0.00004	3	DB04069,DB04395,DB07444	5,6-Dihydro-Benzo[H]Cinnolin-3-Ylamine
SERPINA1	0.9062(0.8370-0.9811)	0.01504	3	DB01998,DB05481,DB03345	Recombinant alpha 1-antitrypsin,Mercaptoethanol
CANX	0.7301(0.6099-0.8739)	0.00061	2	DB00031,DB00025	Tenecteplase, Antihemophilic factor
CCL2	1.1482(1.0510-1.2544)	0.00221	2	DB01055,DB01406	Mimosine, Danazol

## Discussion

Acute myeloid leukemia (AML) is one of the most prevalent hematological cancers that is characterized by the accumulation of immature clones of myeloid progenitors (49). Patients with AML have benefited from advances in targeted molecular and immunotherapy, but the 5-year prognosis for AML remains unsatisfactory owing to high relapse rates. An accurately predicted prognosis improves the decision-making capacity of the physician to select personalized treatment by stratifying the patient into a high- or low-risk group based on a reliable signature. In this study, OS-related ARGs were identified by using profiles of AML patients, and a signature comprising eight ARGs that can accurately predict the OS of patients was developed. The results of external validation suggest that this signature is a steady and independent predictor for the risk stratification of AML patients. In addition, distinct tumor-immune infiltrating landscapes between the high- and low-risk patients as well as potential druggable ARGs were identified through computational biology.

The complex autophagy-related machinery assembled by dozens of known proteins plays a critical role in maintaining essential cellular homeostasis by removing unfolded, excessive, or aged proteins as well as and organelles damaged through stress (50). The dysregulation of autophagy can be a driver of oncogenic transformation (51). Increased activities related to autophagy in cancer cells, resulting from large ratios of compromised cytosol and organelles that can cause the irreversible collapse of vital cellular functions, have been used in anti-cancer therapies. Many autophagy-related genes and signaling pathways have been shown to be key regulators in tumorigenesis and progression, and have been used to target rapamycin complex 1 (mTORC1) and AMP-activated protein kinase (AMPK) signaling pathways that control the induction of phases in mammals (52). The loss of functional mutations in negative regulators, TSC1, TSC2, and PTEN, are recognized for these signaling pathways. The heterozygosity of Beclin1, a key autophagy gene, can significantly promote the possibility of canceration owing to genomic instability in the context of reduced autophagy (53). Autophagy-related processes have been highlighted in AML, and represent an attractive druggable target. Various molecular targets and chemotherapeutic inhibitors of autophagy have been identified (8). The profiling of autophagy-related genes in AML contributes to finding additional prognostic biomarkers, and stratifying high- and low-risk patients.

In this study, 32 ARGs were found to be significantly associated with the OS of patients using univariate Cox proportional hazards regression, and further protein–protein interaction analysis showed that CASP3 and BECN1 were the leading modules correlated with the other ARGs. Previous studies have shown that some of these nodes are involved in the progression of AML through autophagy modulation (42). For example, CASP3 can control AML1-ETO-driven leukemogenesis in a ULK1-dependent pattern (42), and BECN1 plays a vital role in the initiation and progression of autophagy. Consistently with our observations, the reduced expression of BECN1 was correlated with unfavorable prognoses of AML patients (54). TSC2 has been reported to suppress mTOR signaling *via* phosphorylation and inhibition by AKT (55), while mTOR signaling is associated with neoplastic leukemic proliferation by mediating cellular energy response (56). The roles of some OS-related ARGs, such as BAG3, CANX, ERN1, EEF2, CAPN1, P4HB, CCL2, ITGB4, and FAS, in the regulation of autophagy in AML have not been reported. These ARGs may be important markers in AML as they have been implicated in different cancers (57, 58), while further work is needed to examine the underlying molecular mechanisms. Consistent with previous studies (52), we identified autophagy, p53 signaling, AMPK signaling, and apoptosis as significantly enriched pathways. As an intracellular energy sensor, multiple sites of ULK1 were directly phosphorylated by activated AMPK, and the enhanced activity of ULK1 activated the TSC2, a negative regulator of mTORC1 activity (59). This was in line with the fact that the AMPK signaling pathway plays a crucial role in the positive regulation of autophagic processes.

The identification of gene signatures based on transcriptomic profiles is a promising approach to monitor the prognostic risk of cancers (60). We developed an autophagy-related risk signature here consisting of eight OS-related ARGs to predict patients’ outcomes using LASSO Cox regression analysis. Patients in the high-risk group had significantly shorter OS than those in the low-risk group, even when adjusted for clinical variables by using univariate and multivariate Cox regression analyses. In addition, the signature was validated as an independent predictor on two external AML datasets. The AUC values of the ROC curves for 3-year and 5-year OS were 0.75 and 0.76, respectively. The calibration curve also confirmed its capacity for efficient prediction of patient’s outcome. A nomogram that incorporates risk scores and accessible clinical parameters provided the possibility of individual personalized utility to monitor patient’s prognosis. The predictive performance of our signature is comparable to that of a signature related to six autophagies (61), although it can better reveal the potential landscape for immunoregulatory and promotes the discovery of druggable targets for AML patients.

The differentially expressed genes analyzed in the high- and low-risk groups were significantly enriched in the regulation of immune responses, including neutrophil activation, receptor ligand activity, and chemokine activity, and the main immunity-related pathways, such as acute myeloid leukemia, IL-17 signaling pathway, NF-kappa B, and TNF signaling pathways, confirmed that differentiated immune regulators were involved in these two groups. IL-17 induced the sustained production of inflammatory cytokines, such as TNF-a and IL-6, and chemokines (CXCL1, CXCL2) to promote the pathogenesis of AML (62). Furthermore, IL-17 has been shown to activate some common pro-inflammatory signaling pathways, including NF-kB, JNK/P38/ERK, and PI3K. Inflammation can cause immune cells to assemble at the site of a tumor to fight against leukemic cells. An increasing number of pre-clinical studies have shown that tumor-infiltrating lymphocytes (TILs) have a major influence on disease progression and therapeutic response in many cancers (63, 64). The increased infiltration by cytotoxic T cells, memory T cells, and T helper cells is associated with extended predicted survival (65). An analysis of the tumor-infiltrating immune cells showed significantly decreased abundance of CD8+ T cells, resting and activated NK cells, and enhanced rates of resting and activated CD4+ T cells, regulatory T cells, and gamma delta T cells in the high-risk group. This suggests that a strong immunosuppressive microenvironment, featuring immune checkpoint inhibitors, in high-risk patients might lead to a poor response to immunotherapies.

Most patients with AML exhibit resistance to conventional chemotherapy, especially older patients who cannot endure intensive chemotherapy. In such cases, targeting molecular inhibitors combined with therapy offers promising prospects for treatment (66). The levels of expression of CD47, CD33, DOT1L, and IDH2 were negatively correlated with the signature-defined risk score, and patients might respond poorly to inhibitors targeting these genes but might benefit from the blockade of CTLA4 and MDM2. Thus, the autophagy-related signature can reflect the status of immunity of patients with AML and highlight potential immunotherapeutic implications while the underlying mechanisms need to be investigated.

Drug repurposing contributes to the identification of additional uses for approved or experimental chemicals that can accelerate the development of new drugs (67). OS-related ARGs were employed here to explore potential therapeutic candidates by calculating protein-drug interactions in the DrugBank database. A total of 32 druggable chemicals were retrieved to target eight ARGs. The results showed that a Caspase-3 deficiency impairs the self-renewal of leukemic stem cells and delays AE9a-induced leukemogenesis through autophagy by regulating the cleavage of ULK1. This suggests that Caspase-3 has multiple roles in the hematopoietic development and pathogenesis of AML (40). Eleven drugs were obtained to potentially target Caspase-3. For example, minocycline has been reported to induce apoptosis in patients of acute lymphoblastic leukemia, and alleviate harm to human peripheral blood lymphocyte cells (68). This indicates that it might have an effect on AML. Resistant AML cells frequently have deficiencies in the diphthamide synthesis pathway that impairs the ability of tagraxofusp to ADP-ribosylate cellular targets. This is owing to the reduced expression of DPH1, which encodes a diphthamide pathway enzyme, through DNA CpG methylation (69). Diphthamide that targets eEF2 might be a candidate drug for AML (70). The correlation between these ARGs and drugs needed to be investigated in future work.

This study conducted a systematic analysis of autophagy-related transcriptomic profiling and developed a risk prognostic signature based on the survival-related ARGs in AML patients. There remain several limitations that should be taken into consideration when interpreting the findings, however. The enrolled ARGs were identified from the available evidence of their involvement in disease progression, but prospective data are needed to verify their clinical value. The signature was developed and validated by using retrospective, publicly accessible datasets, and requires independent external validation to assess its potential clinical relevance.

## Conclusions

Our study established a prognostic autophagy-related signature comprising eight ARGs for OS prediction in AML patients. The signature was found to be independently associated with OS in the training and validation cohorts. The distinct molecular landscape defined by it, including the pathways, immune infiltration, correlation between targeted therapies, and potential druggable targets, was systematically explored. The underlying molecular mechanisms require further experimental investigation.

## Data Availability Statement

The original contributions presented in the study are included in the article/**Supplementary Material**, further inquiries can be directed to the corresponding authors.

## Author Contributions

Conceptualization and design: DF and HJ. Data acquisition: BZ, YZ, JX, and SW. Methodology: BZ and DF. Data analysis and interpretation: DF and BZ. Writing (original draft): BZ and DF. Writing (review and editing): DF, WN, and HJ. All authors contributed to the article and approved the submitted version.

## Funding

This study was supported by the Science and Technology Research Project of the Jiangxi Province Department of Education (GJJ201837), and the Natural Science Foundation of Jiangxi Province (20192BAB215001).

## Conflict of Interest

The authors declare that the research was conducted in the absence of any commercial or financial relationships that could be construed as a potential conflict of interest.

## Supplementary Material

Supplementary Figure 1 General analysis workflow of this study.

Supplementary Figure 2 Identification of key variables *via* LASSO regression analysis. **(A)** One thousand-fold cross-validation for variable selection in LASSO regression. **(B)** LASSO coefficients of key autophagy-related genes. Each curve represents an autophagy-related gene.

Supplementary Figure 3 Expression of the model genes in the high- and low-risk groups (*P* < 0.05). **(A)** BAG3, **(B)** DAPK1, **(C)** CALCOCO2, **(D)** CAMKK2, **(E)** CANX, **(F)** P4HB, **(G)** TSC2, **(H)** ULK1.

Supplementary Figure 4 Multiple AUCs of risk score and clinical parameters. **(A)** Multiple AUCs for 1 year. **(B)** Multiple AUCs for 3 years. **(C)** Multiple AUCs for 5 years.

Supplementary Figure 5 Gene set enrichment analysis of autophagy-related signature in high- and low-risk groups.

Supplementary Figure 6 Tumor-immune microenvironment analysis of the high- and low-risk groups in two validation sets. **(A)** GSE12417. **(B)** TCGA. The blue violin reflects the low-risk group and the red violin represents the high-risk group.

Supplementary Figure 7 The expression analysis of exhausted T cells markers in high- and low-risk group. **(A)** GZMB. **(B)** IFNG. **(C)** PRF1. **(D)** TNF.

Supplementary Table 1 The characteristics of samples used in this study.

## Abbreviations

AML, Acute Myeloid Leukemia; DEGs, Differentially Expressed Genes; ARGs, Autophagy-related Genes; TCGA, The Cancer Genome Atlas; GEO, Gene Expression Omnibus; RMA, Robust Multi-array Average; HADb, Human Autophagy Database; TRRUST, Transcriptional Regulatory Relationships Unraveled by Sentence-based Text mining; PPI, Protein–Protein Interaction; MCODE. Molecular Complex Detection; AUC, Area Under Curve; ROC, The Receiver Operating Characteristics Curve; GSEA, Gene Set Enrichment Analysis; TFs, Transcription Factors; BP, Biological Process; CC, Cellular Component; MF, Molecular Function; KEGG, Kyoto Encyclopedia of Genes and Genomes; MSigDB, Molecular Signatures Databases; AIC, Akaike Information Criterion; OS, Overall Survival; PD-1, Programmed Cell Death Ligand; FLT3, FMS-like Tyrosine Kinase 3; CTLA4, Cytotoxic T-Lymphocyte-associated Protein 4.
