# Supplementary figures and images for: Prognosis and Characterization of Immune Microenvironment in Acute Myeloid Leukemia Through Identification of an Autophagy-Related Signature
Source: Front Immunol. 2021 May 31;12:695865. doi: 10.3389/fimmu.2021.695865 (PMC8200670; doi:10.3389/fimmu.2021.695865)

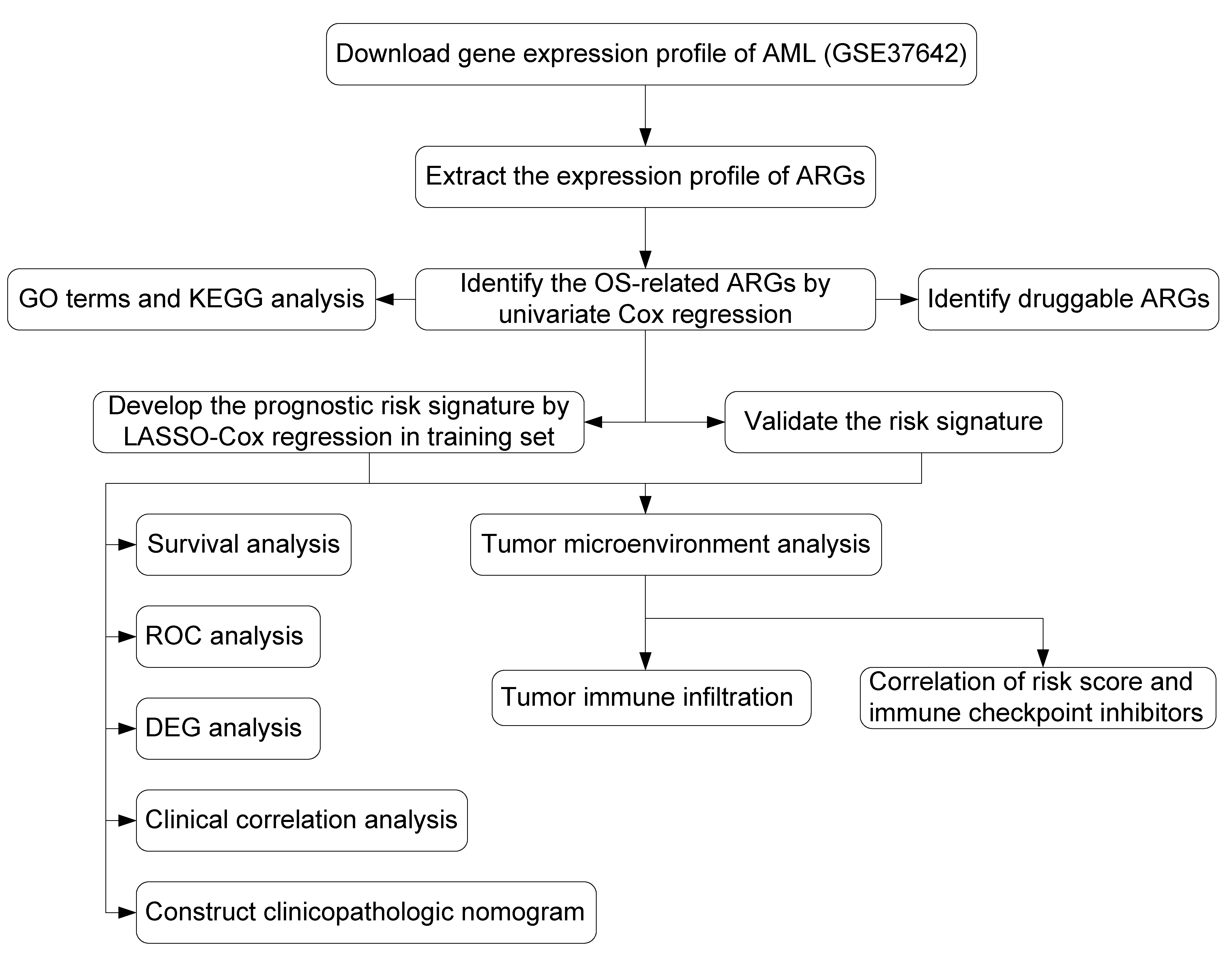

Supplement: Supplementary Figure 1 — General analysis workflow of this study. [file Image_1.tif]

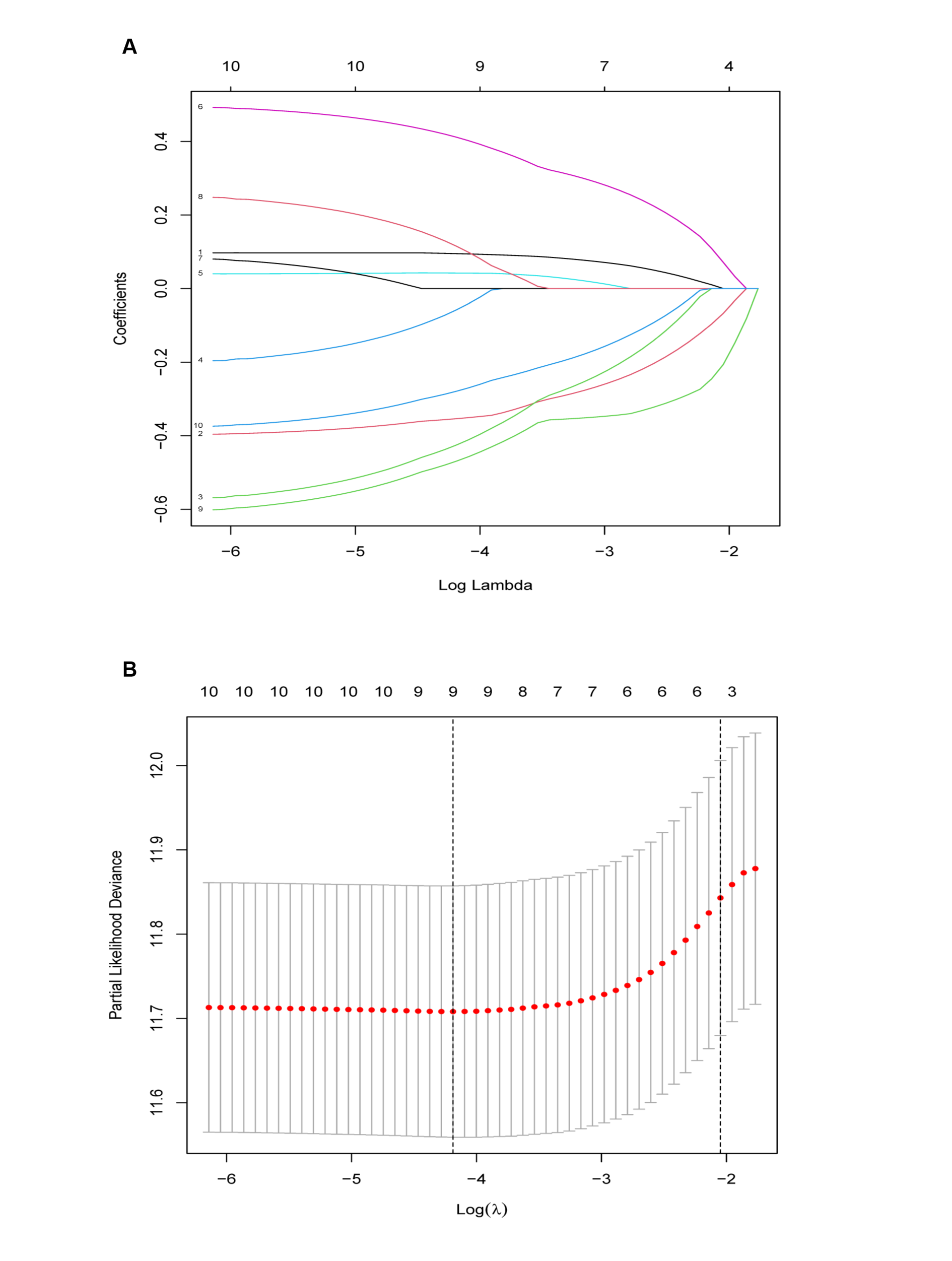

Supplement: Supplementary Figure 2 — Identification of key variables via LASSO regression analysis. (A) One thousand-fold cross-validation for variable selection in LASSO regression. (B) LASSO coefficients of key autophagy-related genes. Each curve represents an autophagy-related gene. [file Image_2.tif]

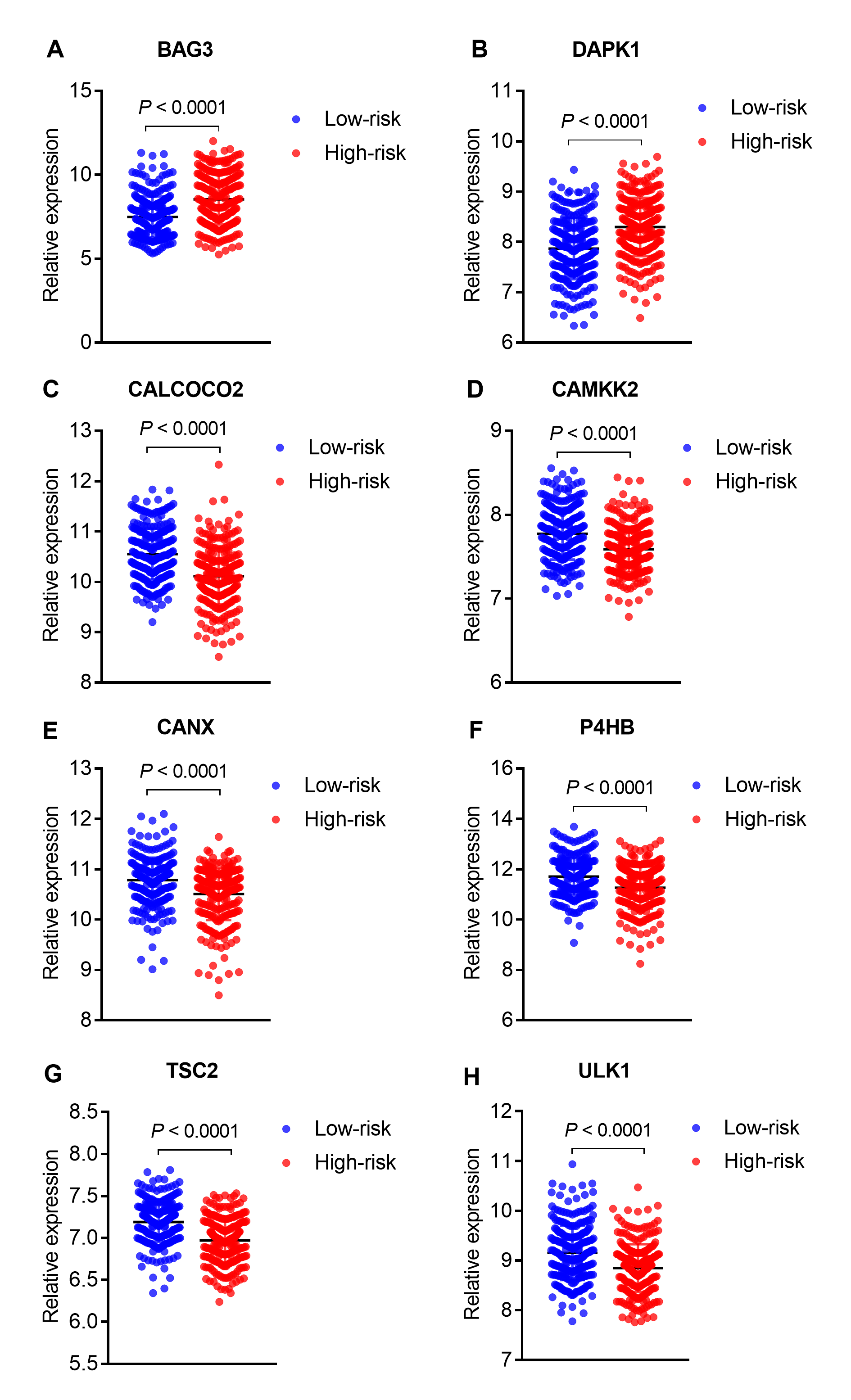

Supplement: Supplementary Figure 3 — Expression of the model genes in the high- and low-risk groups (P < 0.05). (A) BAG3, (B) DAPK1, (C) CALCOCO2, (D) CAMKK2, (E) CANX, (F) P4HB, (G) TSC2, (H) ULK1. [file Image_3.tif]

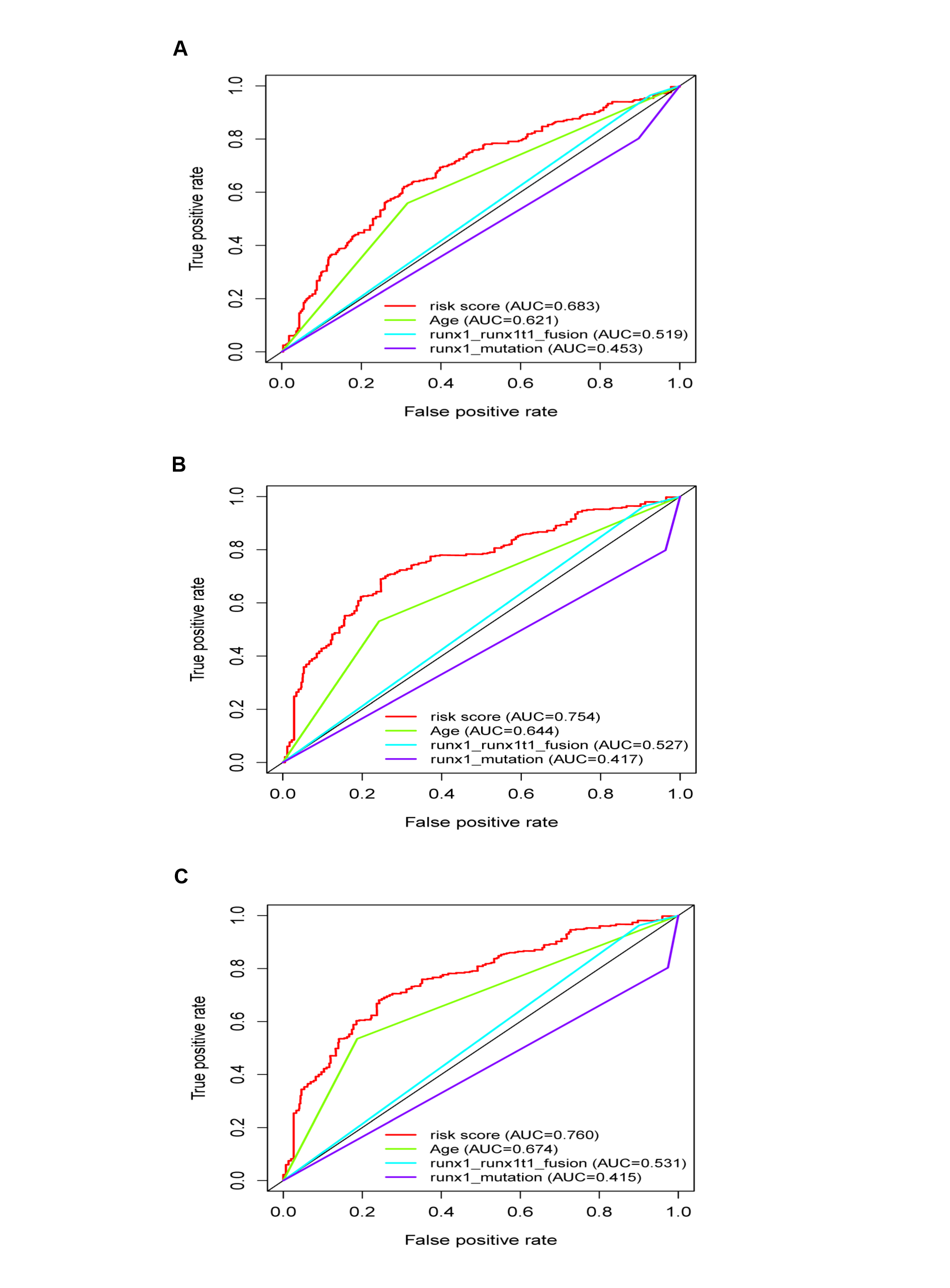

Supplement: Supplementary Figure 4 — Multiple AUCs of risk score and clinical parameters. (A) Multiple AUCs for 1 year. (B) Multiple AUCs for 3 years. (C) Multiple AUCs for 5 years. [file Image_4.tif]

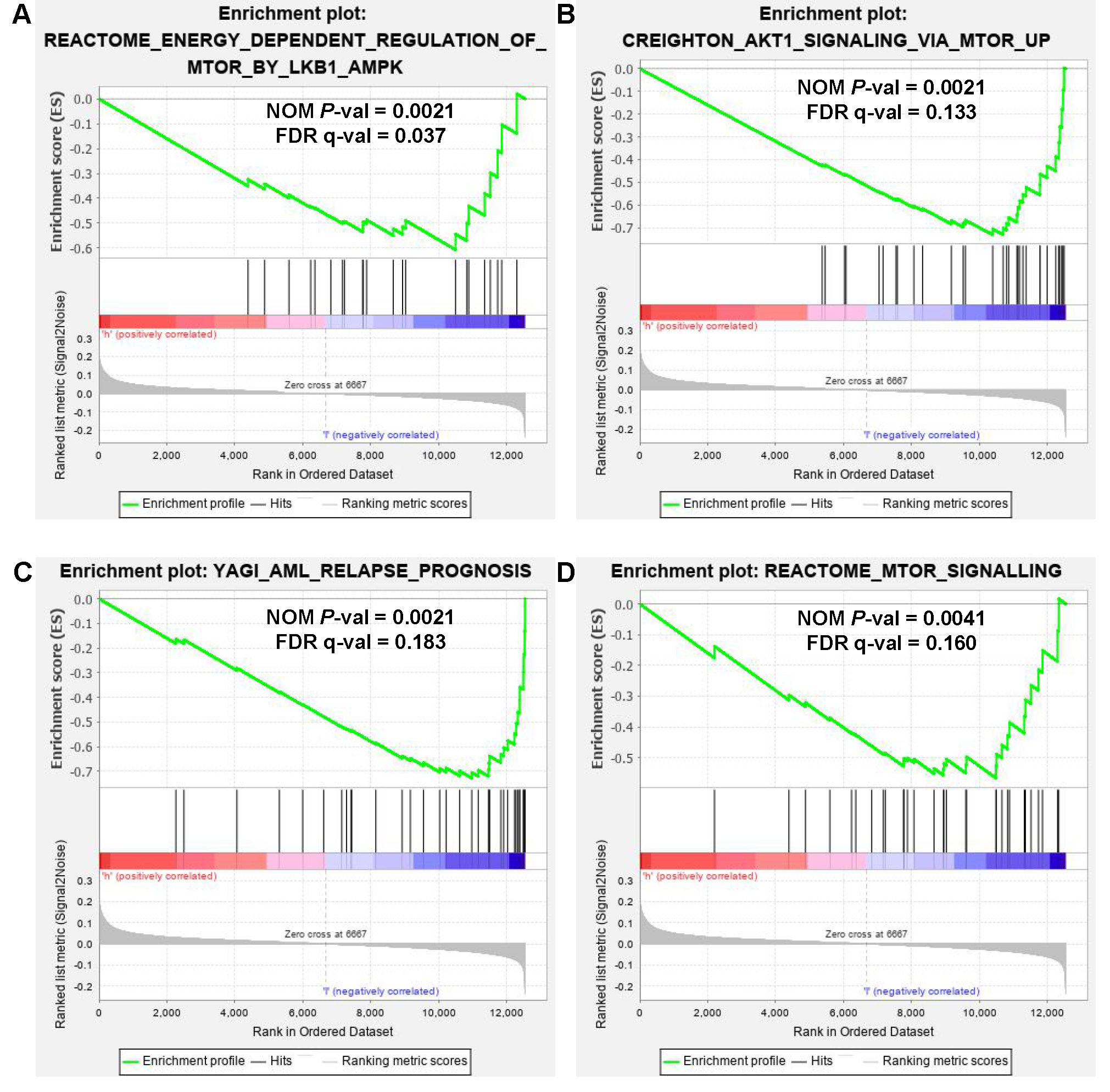

Supplement: Supplementary Figure 5 — Gene set enrichment analysis of autophagy-related signature in high- and low-risk groups. [file Image_5.tiff]

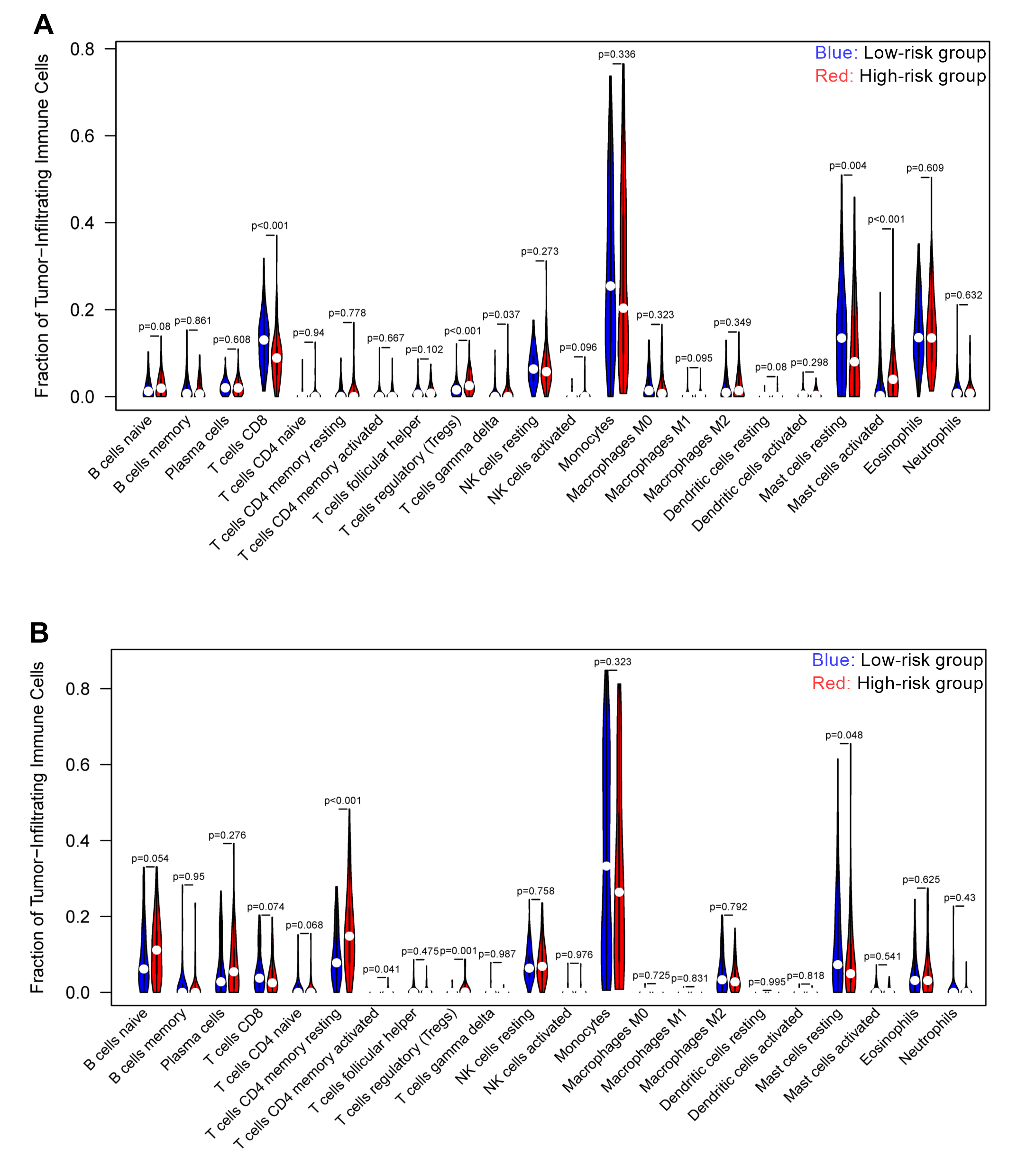

Supplement: Supplementary Figure 6 — Tumor-immune microenvironment analysis of the high- and low-risk groups in two validation sets. (A) GSE12417. (B) TCGA. The blue violin reflects the low-risk group and the red violin represents the high-risk group. [file Image_6.tif]

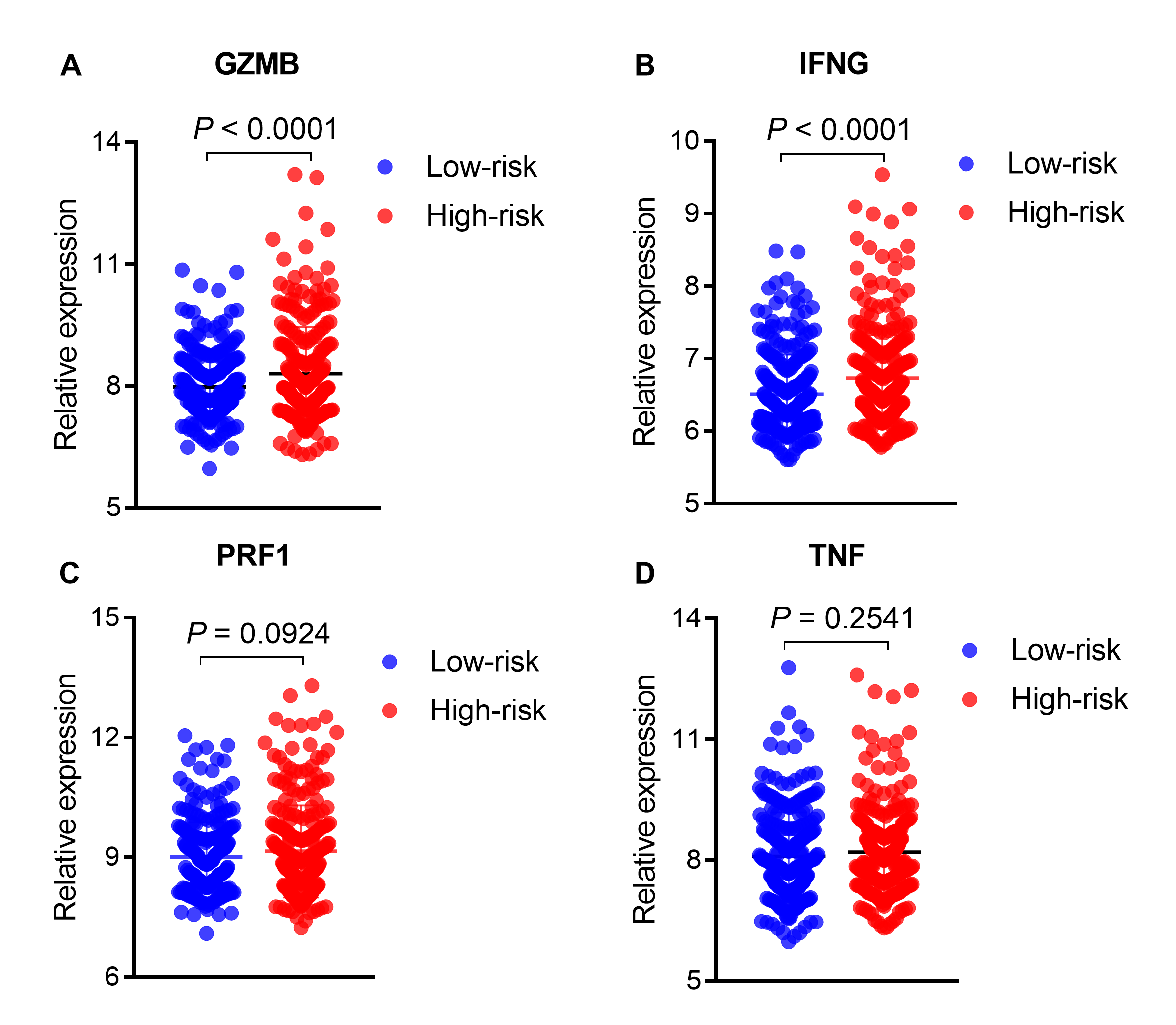

Supplement: Supplementary Figure 7 — The expression analysis of exhausted T cells markers in high- and low-risk group. (A) GZMB. (B) IFNG. (C) PRF1. (D) TNF. [file Image_7.tif]
